# The landscape of the COVID-19 pandemic in Poland emerging from epidemiological and genomic data

**DOI:** 10.1038/s41598-024-65468-5

**Published:** 2024-06-22

**Authors:** Barbara Mirska, Michal Zenczak, Katarzyna Nowis, Ireneusz Stolarek, Jan Podkowiński, Magdalena Rakoczy, Małgorzata Marcinkowska-Swojak, Natalia Koralewska, Paweł Zmora, Elżbieta Lenartowicz Onyekaa, Marcin Osuch, Katarzyna Łasińska, Jadwiga Kuczma-Napierała, Marcelina Jaworska, Łukasz Madej, Marzena Ciechomska, Aleksander Jamsheer, Krzysztof Kurowski, Marek Figlerowicz, Luiza Handschuh

**Affiliations:** 1 Institute of Bioorganic Chemistry Polish Academy of Sciences, Poznan, Poland; 2 Provincial Sanitary and Epidemiology Station in Poznan, Poznan, Poland; 3 Baptism of Poland Memorial Hospital, Gniezno, Poland; 4 Regional Science and Technology Center, Podzamcze, Poland; 5 National Institute of Geriatrics, Rheumatology and Rehabilitation, Warsaw, Poland; 6 Department of Medical Genetics, Poznan University of Medical Sciences, Poznan, Poland; 7 Centers for Medical Genetics GENESIS, Poznan, Poland

**Keywords:** COVID-19, SARS-CoV-2, Pandemic, Epidemiology, Genomics, Haplotype network, Computational biology and bioinformatics, Genetics, Diseases

## Abstract

The COVID-19 pandemic has profoundly affected all aspects of our lives. Through real-time monitoring and rapid vaccine implementation, we succeeded in suppressing the spread of the disease and mitigating its consequences. Finally, conclusions can be summarized and drawn. Here, we use the example of Poland, which was seriously affected by the pandemic. Compared to other countries, Poland has not achieved impressive results in either testing or vaccination, which may explain its high mortality (case fatality rate, CFR 1.94%). Through retrospective analysis of data collected by the COVID-19 Data Portal Poland, we found significant regional differences in the number of tests performed, number of cases detected, number of COVID-19-related deaths, and vaccination rates. The Masovian, Greater Poland, and Pomeranian voivodeships, the country’s leaders in vaccination, reported high case numbers but low death rates. In contrast, the voivodeships in the eastern and southern parts of Poland (Subcarpathian, Podlaskie, Lublin, Opole), which documented low vaccination levels and low case numbers, had higher COVID-19-related mortality rates. The strong negative correlation between the CFR and the percentage of the population that was vaccinated in Poland supports the validity of vaccination. To gain insight into virus evolution, we sequenced more than 500 genomes and analyzed nearly 80 thousand SARS-CoV-2 genome sequences deposited in GISAID by Polish diagnostic centers. We showed that the SARS-CoV-2 variant distribution over time in Poland reflected that in Europe. Haplotype network analysis allowed us to follow the virus transmission routes and identify potential superspreaders in each pandemic wave.

## Introduction

Coronavirus disease 2019 (COVID-19), caused by severe acute respiratory syndrome coronavirus 2 (SARS-CoV-2), emerged in November 2019 in Wuhan, China, and quickly spread worldwide. Between December 2019 and December 2022, the novel coronavirus infected more than 660 million people and caused nearly 6.69 million deaths worldwide^1–3^. Approximately 36% of the world’s cases and 29% of the total deaths occurred in Europe^2^. Poland's first SARS-CoV-2 infection case was confirmed and reported on March 4, 2020. As of January 1, 2023, with 6.3 million cases of COVID-19, Poland was ranked 8th in Europe and 21st in the world^2,3^. Additionally, with 118.5 thousand reported deaths, Poland was ranked 7th and 16th in Europe and the world, respectively. Therefore, Poland may be considered one of the countries most seriously affected by the COVID-19 pandemic.

The appearance of COVID-19 in Poland caused the implementation of restrictions according to WHO recommendations, including lockdowns during the first wave of the pandemic. From March 20, 2020, to May 15, 2022, the epidemic was in force in Poland, and restrictions were temporarily suspended or restored depending on the number of active cases^4^. Once vaccines were developed and approved, the Polish government announced the National COVID-19 Vaccination Program and recommended vaccination as an effective method for preventing SARS-CoV-2 infection^5^. Mathematical modeling confirmed the validity of this strategy^6^. However, despite nationwide campaigns and educational programs promoting vaccination, the general attitude toward vaccination in Poland was cautious^7,8^. As a result, on January 1, 2023, only 56.76% of the Polish population was fully vaccinated^3,9^. For comparison, at the same time, the world average was 63.39%, while for Europe and the European Union, these values were even greater (66.90% and 72.82%, respectively)^3,9^.

The development of tests and vaccines would not be possible without knowledge about the SARS-CoV-2 sequence and structure. Chinese scientists published the first whole-genome sequence of the novel zoonotic betacoronavirus in January 2020^10^. To follow the evolution of the virus, mass sequencing of samples from infected patients began. At the beginning of 2023, almost 15 million SARS-CoV-2 whole-genome sequences were collected by the Global Initiative on Sharing All Influenza Data (GISAID, www.gisaid.org)^11^. Corresponding repositories were established locally, e.g., the European COVID-19 Data Platform (www.covid19dataportal.org), operated by the European Commission and EMBL's European Bioinformatics Institute (EMBL-EBI)^12^. As a part of the EMBL-EBI initiative, we (the Institute of Bioorganic Chemistry Polish Academy of Sciences (IBCH PAS) and the affiliated Poznan Supercomputing and Networking Center (PSNC)) launched the COVID-19 Data Portal Poland (https://dataportal.covidhub.psnc.pl)^13^. This platform includes databases and tools supporting researchers in sharing and visualizing data associated with SARS-CoV-2 and COVID-19 in Poland.

Here, for the first time, we present a comprehensive analysis of the situation in Poland over two years of the pandemic across the country and against a worldwide background. Taking advantage of the COVID-19 Data Portal Poland, we retrospectively traced the development of the pandemic in our country. In addition, we analyzed nearly 80 thousand SARS-CoV-2 genome sequences available in the GISAID database, including more than 500 genomes sequenced in our laboratory.

## Methods

### Sample collection

Postdiagnostic nasopharyngeal samples (1027) and previously extracted RNA samples (81) were obtained from the Provincial Sanitary-Epidemiological Station and Centers for Medical Genetics GENESIS in Poznan, Baptism of Poland Memorial Hospital in Gniezno, the Regional Science and Technology Center in Podzamcze and the National Institute of Geriatrics, Rheumatology and Rehabilitation in Warsaw. The study conformed to the ethical guidelines of the World Medical Association Declaration of Helsinki. Because the biological material came from postdiagnostic waste and the samples were anonymized, it was not possible to obtain consent from individual people. However, appropriate approval was obtained from the Bioethics Committee at the Poznan University of Medical Sciences to use archival material (No. 457/20 from 17 June 2020). The data deposited in the databases did not contain any information based on which sample donors could be identified.

### Epidemiological data analysis

Epidemiological data and worldwide statistics at the country level were retrieved from public repositories, i.e., Our World In Data^3^ and Worldometer^2^. Regional data for Poland were downloaded from the COVID-19 Data Portal Poland^13^.

*Viral RNA isolation*. Total RNA from nasopharyngeal swabs was extracted with a CoV RNA kit (A&A Biotechnology, Poland) in the BSL-2 chambers of the Laboratory of Cell and Tissue Culture, IBCH PAS. Before library preparation, the samples were additionally checked for viral load via RT‒qPCR of the ORF1a/b and S genes via the MediPAN-2G COVID test (IBCH PAS/Medicofarma, Poland).

*Next-generation sequencing*. RNA was reverse transcribed to complementary DNA (cDNA) with SuperScript IV Reverse Transcriptase (Thermo Fisher Scientific, MA, USA). Second strand synthesis was performed with the NEBNext Ultra II Non-Directional RNA Second Strand Synthesis Module (New England Biolabs). One nanogram of dsDNA was used for library preparation with the TruePrep DNA Library Prep Kit V2 for Illumina (1 ng) (Vazyme). Indexed DNA libraries were pooled (4–6 samples) and subjected to probe-based enrichment with a myBaits Expert SARS-CoV-2 Panel (Arbor Biosciences). Library quantitation was performed with a Qubit DNA HS Assay (Thermo Fisher Scientific). For the library quality check, the Bioanalyzer 2100 and DNA 1000 Kit or DNA High Sensitivity Kit (Agilent Technologies, CA, USA) were used according to the manufacturer’s instructions. Libraries were sequenced on an Illumina NextSeq 550 platform using paired-end reads and a NextSeq 500/550 High Output Kit v2.5 (300 cycles) (Illumina, San Diego, CA, USA). Up to 96 samples were sequenced per run, resulting in an average of 10.5 million (median 3.15 million) raw reads and an average coverage of 3233x (median 370x) per sample. The raw fastQ files were aligned to the SARS-CoV-2 reference sequence (*NC_045512.2*)^14^ with BBMap^15^. BAM files were further processed with BBTools, SAMtools and BCFtools to create final consensus sequences^16^.

*Analysis of SARS-CoV-2 genome sequencing data.* SARS-CoV-2 sequences dated up to May 23, 2022, were downloaded from GISAID (11) (EPI_SET_231114ua). First, the search criteria were limited according to host (human), location (Poland), and complete genome sequence (> 2900 nt). Next, we excluded low-coverage entries with more than 5% unknown nucleotides (N). We used the same inclusion criteria for our sequencing data. Then, we performed redundant metadata cleanup and excluded repeated data records, nonhuman origin records, and records without a complete subset of metadata for a final number of 79,194 (78,674 from GISAID and 520 from our laboratory).

*Haplotype network.* A total of 79,194 SARS-CoV-2 sequences were divided into five pandemic waves. Due to the large number of sequences representing the 3rd, 4th and 5th waves, we subsampled the raw data for those waves with the Genome-Sampler tool^17^. The first filtering step involved selecting genomes across time, with the default parameters of seven genomes per seven-day interval. The second filter was diversity-based; the sequences were clustered at the 99.00% identity threshold, and the centroid sequence from each cluster was selected as a representative. Finally, a minor allele frequency (MAF) filter of 5% was applied to exclude rare variants. Then, the consensus sequences were created and submitted for haplotype network analysis. Rapid calculation of the full-length MSAs of closely related viral genomes was performed with MAFT-7.490 using the default parameters –6merpair, –keeplength, and –addfragments^18^. The haplotype network was created in the R package (R 4.2.0) as follows^19^. The sequences were trimmed with the ips package. For haplotype extraction and network construction, the pegas package was applied^20^. Finally, the network was visualized using igraph (layout_with_graphopt-force-directed layout algorithm used for scaling the graph)^21,22^.

*Statistical data visualization.* Maps and selected plots presenting the epidemiology data were retrieved from the COVID-19 Data Portal Poland^13^ and Our World In Data Portal^3^. Other graphs for data visualization were generated using the ggplot2 package^23^ in R 4.2.0 and OriginPro 2021 9.8.0.200. The phylogenetic tree and variant frequencies of SARS-CoV-2 worldwide and in Europe were visualized via Nextstrain (https://gisaid.org/phylodynamics/global/nextstrain/) used under the CC-BY-4.0 license^24^.

### Ethics approval and consent to participate

Patient consent was not applicable because only postdiagnostic archival and anonymized material was used in the study. Appropriate approval was obtained from the Bioethics Committee at the Poznan University of Medical Sciences (No. 457/20 from 17 June 2020).

## Results

To compare the pandemic situation in Poland with those of other countries, we analyzed epidemiological data from Worldometer^2^, Our World In Data Portal^3^ and the COVID-19 Data Portal Poland^13^, as summarized in Supplementary Table 1.

### COVID-19 cases and tests in Poland compared to other countries

**Figure 1:**
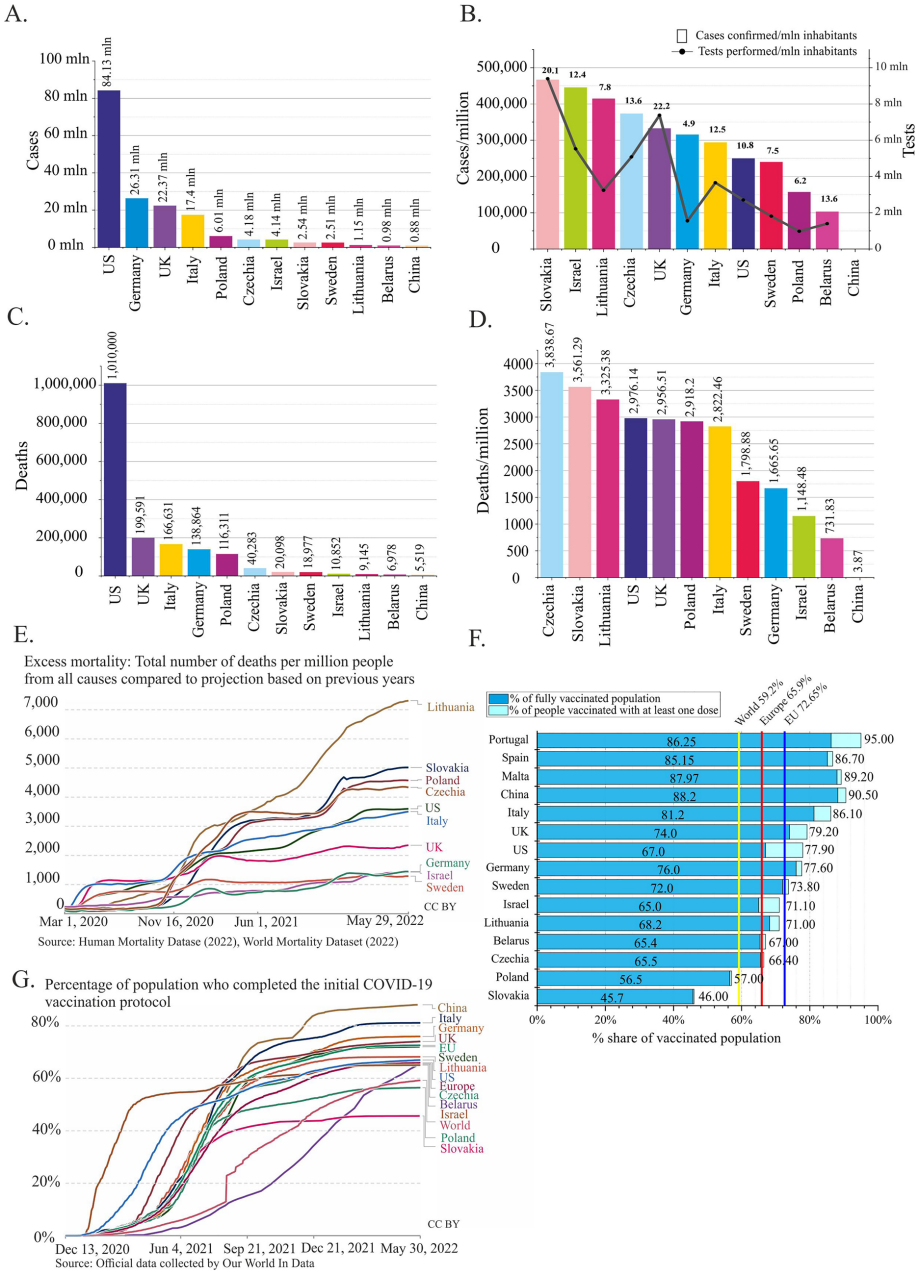
Statistics of COVID-19 in Poland compared to those in other countries (from January 22, 2020, to May 30, 2022; source: Our World In Data database). (**A**) Total number of reported cases of SARS-CoV-2 infection per country. (**B**) Number of cases and tests performed in each country, normalized per 1 million people. The ratio between the number of tests and the number of cases is indicated above each bar. In China, the number of cases/million people is 619.78, and data on the number of tests are unavailable. (**C**) The total number of deaths due to COVID-19 in selected countries. The exact number is indicated above each bar. (**D**) The number of deaths due to COVID-19 in selected countries normalized per 1 million people. The exact number is indicated above each bar. (**E**) Excess mortality. Belarus and China were excluded due to incomplete data. (**F**) Percentage of the population that is fully vaccinated in each country (rounded to the decimal). (**G**) Timeline showing the proportion of individuals who completed the initial COVID-19 vaccination protocol. Graphs E, F, and G were generated by Our World In Data (www.ourworldindata.org) under the CC BY 4.0 license.

Poland had almost 14 times fewer COVID-19 cases than the US, nearly four times fewer than Germany and the UK, and 2.8 times fewer than Italy; Poland also had 1.5 times more cases than Israel and Czechia and approximately 2.4 times more than Slovakia and Sweden (Fig. 1A)^3^. Because these data are not conclusive without adjustment for the country's population, we compared the number of COVID-19 cases per million inhabitants in the same countries (Fig. 1B). In Poland, this number exceeded 150 thousand. In the US and Sweden, it was 1.6 times greater, whereas in Germany and Italy, it was two times greater. Interestingly, very high numbers were recorded in Slovakia (> 460 thousand), Israel (> 440 thousand), and Lithuania (> 410 thousand).

As the observed differences might be influenced by the number of tests carried out in individual countries, we calculated the ratio of the number of tests to the number of COVID-19 cases per million people (Fig. 1B, Supplementary Table 1). Poland, with a ratio of 6.2, Germany (4.9), Sweden (7.5), and Lithuania (7.8) were among the countries with the lowest ratios. Notably, in the countries that implemented massive testing strategies, this ratio exceeded 20 (the UK and Slovakia).

### Mortality due to COVID-19 in Poland compared to other countries

Nearly one-sixth of total COVID-19-related deaths were reported in the US (> 1 million deaths; February 2023)^2^. Over the same period, in Poland, nearly 8.6 times fewer (< 120 thousand) deaths due to COVID-19 were registered^13^. Poland reported more than 2.9 times more deaths than Czechia, 5.9 times more deaths than Slovakia, 12.5 times more deaths than Lithuania, and 16.7 times more deaths than Belarus (Fig. 1C). Germany was the only neighboring country of Poland with a greater total number of deaths. Unsurprisingly, there was a very strong correlation between the total number of cases and number of deaths reported in each country (Pearson’s r = 0.98, *p* = 1.66E−8; Supplementary Fig. 1A).

Unexpectedly, a comparison of the number of COVID-19-related deaths normalized per million people indicated that the mortality rate in Poland was similar to that in the US, UK, and Italy and was 1.75 times greater than that in Germany (Fig. 1D). Even higher mortality rates were noted in Czechia, Slovakia, and Lithuania. We found a moderate correlation between the normalized values of COVID-19 cases and deaths in each country (Pearson’s r = 0.64, *p* = 0.024; Supplementary Fig. 1B).

To objectively compare mortality rates, we calculated the case fatality rate (CFR) for each country (Supplementary Table 2). The CFR indicates the relationship between the total number of deaths due to COVID-19 and the number of diagnosed cases and is expressed as a percentage. Surprisingly, among the analyzed countries, the highest CFR was observed in Poland (1.94%). This result was significantly greater than that of the global CFR (1.20%), twice as high as that of the European CFR (0.95%), and almost three times greater than that of the average EU CFR (0.78%). A CFR equal to the global average was noted in the US (1.20%), whereas the lowest was in Israel (0.26%).

To estimate the impact of COVID-19 on overall mortality in each country, we also analyzed the excess mortality value, calculated as the difference between the cumulative number of deaths (per million) and the number of deaths predicted based on previous years. The greatest number of excess deaths were observed in Lithuania, Slovakia, Poland, and Czechia (Fig. 1E). The shapes of the curves plotted in Fig. 1E indicate the times of rapid increase in excess mortality, reflecting the pandemic waves (spring 2020, autumn 2020, and winter 2021/2022).

### Vaccination against COVID-19 in Poland compared to other countries

As of May 30, 2022, on average, 59.2% of the world’s population has been fully vaccinated against SARS-CoV-2^3,9^. At the same time, the European average was 65.9%, and the EU average was 72.6%. In Europe, Malta (88%), Portugal (86%), and Spain (85%) were the leaders in vaccination. The vaccination rate achieved by Poland (56.5%) was below the global average (Fig. 1F)^5^. Among the neighboring countries of Poland, a lower percentage was noted only in Slovakia (45.7%). In Czechia, Belarus, Lithuania, and Germany, vaccination rates were much greater (66.5%, 65.4%, 68.2%, and 76%, respectively) (Fig. 1F). Notably, very high vaccination rates were observed in China (88.2%) and Italy (81.2%), which were severely affected by COVID-19 at the beginning of the pandemic (Fig. 1F).

By analyzing the vaccination timeline, we concluded that most populations were vaccinated shortly after the vaccination programs started, i.e., between April and August 2021 (Fig. 1G). Initially, countries such as Israel, the US, and the UK were leaders in vaccination but were eventually surpassed by other countries (e.g., China, Italy, and Germany). In Poland, before the massive vaccination program was launched, particularly vulnerable groups were vaccinated (> 65 years old, teachers, and health care workers). The percentage of people who completed the initial vaccination protocol increased rapidly between May and July 2021, exceeding 40%. After that, the rate of vaccination slowed. We found a moderate negative correlation between the numbers of COVID-19 cases and deaths (normalized per million people) and the percentage of the population vaccinated with at least one dose of COVID-19 vaccine, but the results were not statistically significant (case-vaccination Pearson’s r = − 0.39, *p* value = 0.22; death-vaccination Pearson’s r = − 0.45, *p* value = 0.13; Supplementary Fig. 1C and D, respectively).

### Regional distribution of SARS-CoV-2 infections in Poland

**Figure 2:**
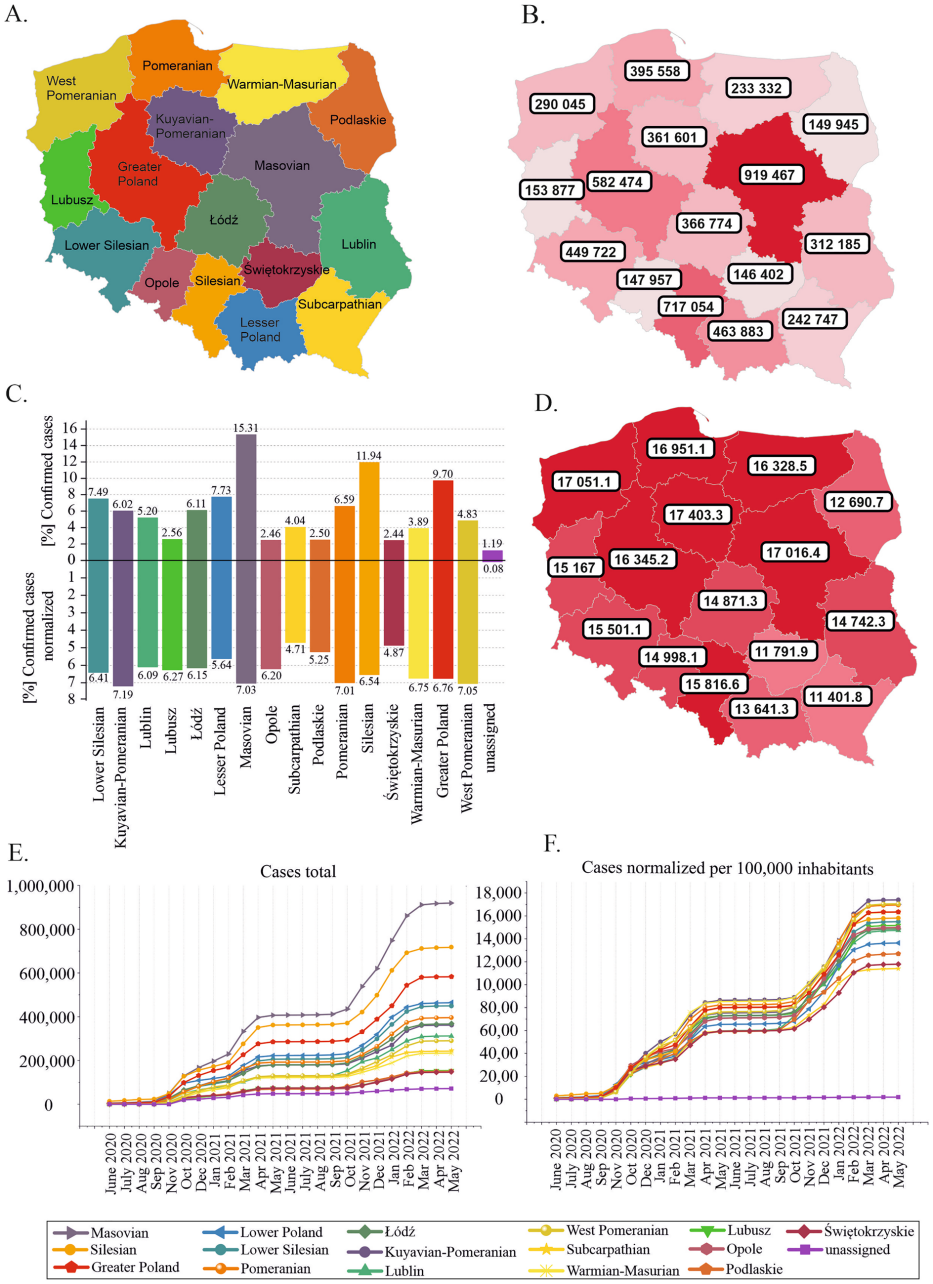
Regional distribution of SARS-CoV-2 infections between June 2020 and May 30, 2022. (**A**) Division of voivodeships in Poland. (**B**) Total number of COVID-19 cases registered in each voivodeship. (**C**) Number of cases in Poland converted to percentages (rounded to hundredths). (**D**) Regional distribution of cases normalized per 100,000 people. (**E**) Growth curve of the number of cases by voivodeship. (**F**) Growth curve of the number of cases normalized per 100,000 population by voivodeship. Maps were downloaded from the COVID-19 Data Portal Poland^13^ (https://covidhub.psnc.pl/eng/mapa/), which utilized OpenStreetMap under the Open Database License, “ODbL” 1.0.

To gain deeper insight into the situation in Poland, we analyzed data collected by the COVID-19 Data Portal Poland^13^. On May 30, 2022, the highest number of cases was reported in the Masovian voivodeship (15.31% of all cases), followed by Silesian (11.94%) and Greater Poland (9.70%), whereas the lowest number was reported in Świętokrzyskie (2.44%), Opole (2.46%), Podlaskie (2.50%), and Lubusz (2.56%) (Fig. 2B,C, Supplementary Table 3A).

Normalization of the case numbers per 100,000 people reduced the differences between voivodeships (Fig. 2C,D), but the differences remained statistically significant (chi-square test value = 3.49E+3, two-tailed *p* value < 0.0001). More SARS-CoV-2 infections were detected in northern and central Poland, particularly in the Kuyavian-Pomeranian (7.19%), West Pomeranian (7.05%), Masovian (7.03%), and Pomeranian (7.01%) voivodeships. The lowest percentages were recorded in Subcarpathian (4.71%) and Świętokrzyskie (4.87%), which are located in southeastern Poland, and Podlaskie (5.25%), which is located in northeastern Poland (Fig. 2C,D, Supplementary Table 3B). A moderate positive correlation was found between population density and the total number of cases in each voivodeship (Pearson’s r = 0.64, *p* = 0.00743; Supplementary Fig. 2A).

The increase in infection rate over time showed a similar trend in all the voivodeships (Fig. 2E, F). The plot indicates several time points when cases increased faster: November 2020-February 2021, February-April 2021, and November 2021-February 2022.

### Regional distribution of COVID-19-related deaths in Poland

**Figure 3:**
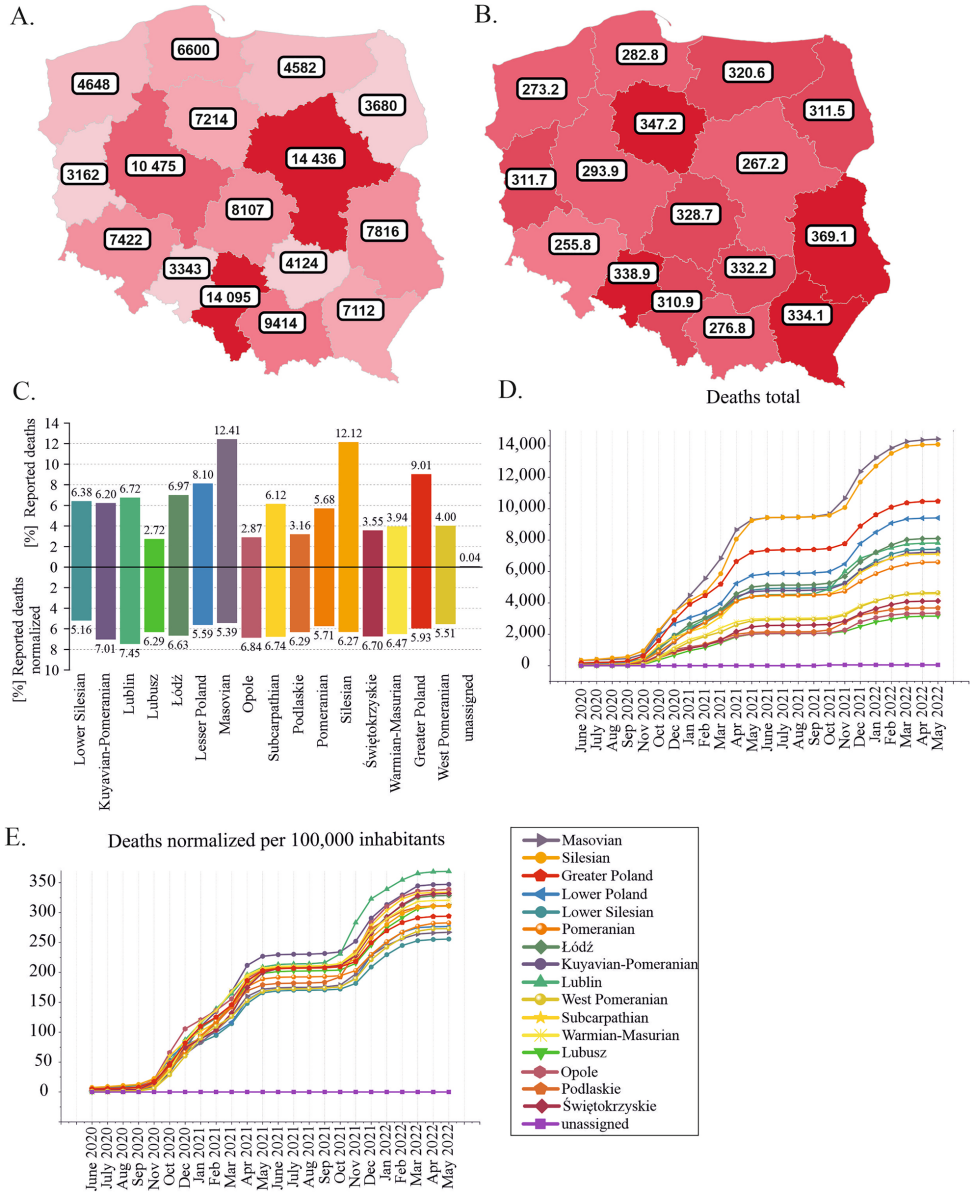
Regional distribution of all COVID-19-related deaths between June 2020 and May 30, 2022. (**A**) Total number of reported deaths in each voivodeship. (**B**) Regional distribution of deaths normalized per 100,000 people. (**C**) Number of deaths converted to percentages (rounded to hundredths). (**D**) Growth curves of the number of deaths by voivodeship. (**E**) Growth curves of the number of deaths normalized per 100,000 people by voivodeship. Maps were downloaded from the COVID-19 Data Portal Poland^13^ (https://covidhub.psnc.pl/eng/mapa/), which utilized OpenStreetMap under the Open Database License, “ODbL” 1.0.

The highest numbers of COVID-19-related deaths were reported in the Masovian (12.41% of all cases in Poland), Silesian (12.12%), and Greater Poland (9.01%) voivodeships (Fig. 3A, C, Supplementary Table 4A). This finding is consistent with the total number of cases reported in these regions (Fig. 2B). The lowest percentages of deaths were recorded in the Lubusz, Opole, Podlaskie, and Świętokrzyskie voivodeships (2.72%, 2.87%, 3.16%, and 3.55%, respectively). Notably, these regions also had the lowest percentage of SARS-CoV-2 infections (Fig. 3C). A very strong correlation was found between the number of cases and number of deaths in each voivodeship (Pearson's r = 0.96, *p* = 8.36E−9; Supplementary Fig. 2B).

The highest CFR values were observed for Subcarpathian (2.92%), Świętokrzyskie (2.81%), and Lublin (2.50%), while the lowest CFR values were observed for Masovian (1.57%), Lower Silesian (1.65%), and Pomeranian (1.67%) (Supplementary Table 5). We found a strong correlation between the number of deaths and population density for each voivodeship (Pearson’s r = 0.73, *p* = 0.00109; Supplementary Fig. 2C) but no correlation between the CFR and population density (Pearson's r = − 0.18, *p* = 0.5; Supplementary Fig. 2D).

Normalization per 100,000 people reduced the differences in the number of COVID-19-related deaths among voivodeships, but the difference remained statistically significant (chi-square test value = 5.02E+01, two-tailed *p* value < 0.0001). However, the ranking changed completely (Fig. 3B,C, Supplementary Table 4B). The highest percentages of population-normalized deaths were recorded in the Lublin (7.45%), Kuyavian-Pomeranian (7.01%), Opole (6.84%) and Subcarpathian (6.74%) voivodeships. In contrast, the lowest percentages were observed for the Lower Silesian (5.16%), Masovian (5.39%), and West Pomeranian (5.51%) voivodeships. The high mortality in the Kuyavian-Pomeranian voivodeship was consistent with the high population-normalized number of cases in this region (Fig. 2C,D), but this relationship was not observed for other voivodeships. For example, Lublin and Opole were in the middle of the ranking of the normalized number of cases, while Subcarpathian was the last in the country (Fig. 2C,D). In contrast, in terms of the normalized number of deaths, these regions were ranked 1st, 3rd, and 4th in the country, respectively (Fig. 3B,C).

Analysis of the increase in the number of deaths over time indicated a similar pattern for all regions (Fig. 3D,E). In general, this increase was consistent with the increase in SARS-CoV-2 infections, reflecting the dynamics of the COVID-19 pandemic in Poland.

### Vaccination in Poland: regional distribution

**Figure 4:**
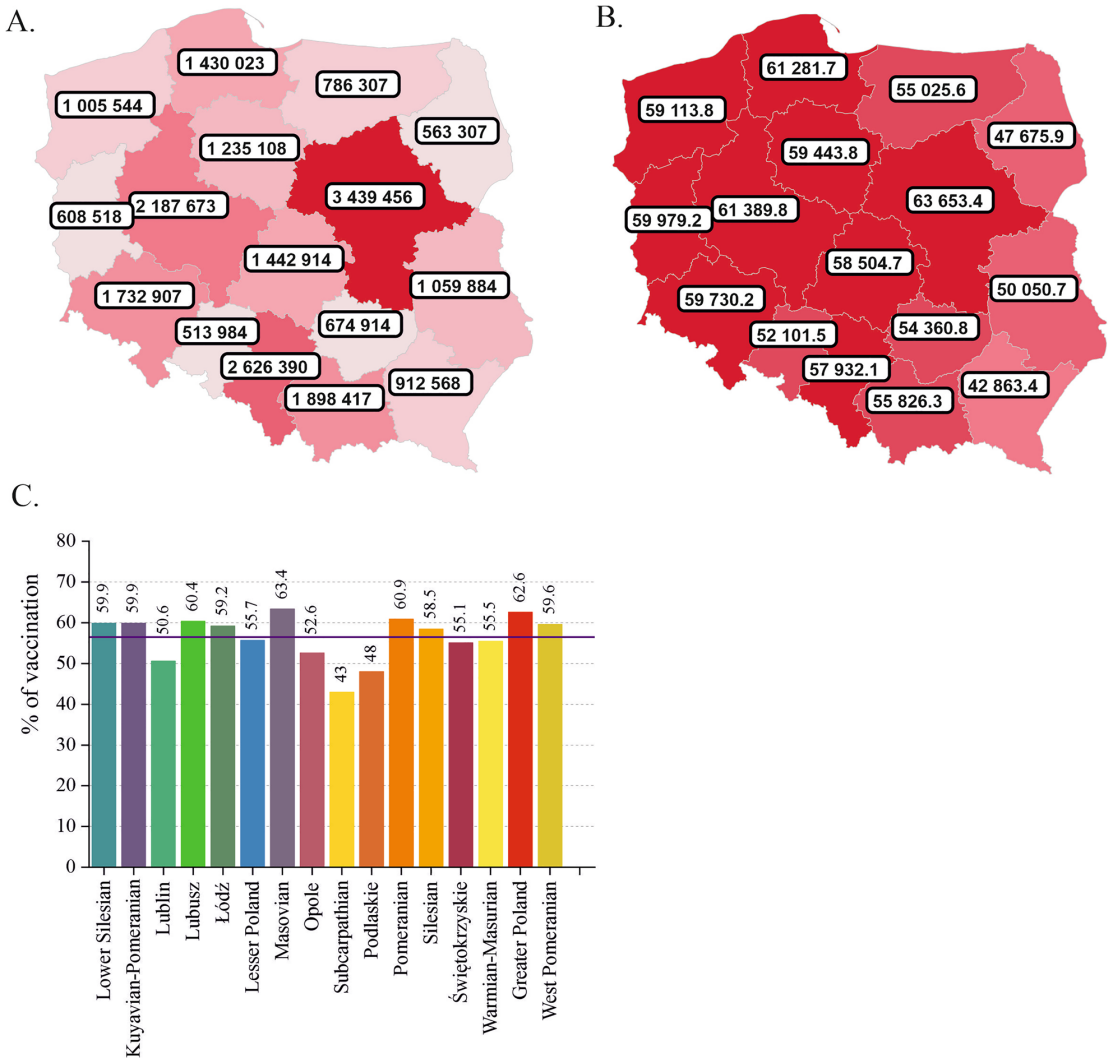
Status of vaccination against COVID-19 in Poland (May 30, 2022). (**A**) Total number of people vaccinated by voivodeship. (**B**) Number of vaccinated people normalized per 100,000 people. (**C**) Vaccination rate expressed as a percentage of the vaccinated population in each voivodeship. The horizontal line represents the national vaccination average of 56.5%. Maps were downloaded from the COVID-19 Data Portal Poland^13^ (https://covidhub.psnc.pl/eng/mapa/), which utilized OpenStreetMap under the Open Database License, “ODbL” 1.0.

In Poland, the highest total numbers of fully vaccinated people were recorded in three voivodeships, namely, Masovian, Silesian, and Greater Poland, while the lowest were recorded in Opole, Podlaskie, Lubusz, and Świętokrzyskie (Fig. 4A). For the numbers of fully vaccinated people normalized per 100,000 people, the leaders were Masovian, Greater Poland, and Pomeranian (Fig. 4B). These three voivodeships had a remarkably high percentage of reported cases (Fig. 2C) but a relatively low death rate (Fig. 3C).

The lowest level of vaccination was recorded in the voivodeships located in the eastern part of the country, namely, Subcarpathian (43%), Podlaskie (48%), and Lublin (50.6%), followed by Opole voivodeship in the south (52.6%) (Fig. 4C). Notably, Subcarpathian and Podlaskie were among the regions with the lowest percentage of cases in the country, while in Lublin and Opole, a moderate percentage of cases was observed (Fig. 2C). However, Lublin, Opole, and Subcarpathian had the highest mortality rates in the country (Fig. 3C). We found a strong negative correlation between the CFR and the percentage of the population that was vaccinated in each voivodeship (Pearson’s r = − 0.83, *p* = 7.04E−5; Supplementary Fig. 2E, Supplementary Table 5).

### Next-generation sequencing of SARS-CoV-2 genomes

In cooperation with six health care and diagnostic institutions in Poland, we collected postdiagnostic samples from SARS-CoV-2-infected people from different regions. In total, over a thousand samples were collected between May 2020 and August 2021, encompassing the 1st and 2nd waves of coronavirus in Poland and the time between the waves. Due to the poor quality and/or quantity of some RNA samples, only 729 were used for library preparation. After the quality assessment, we chose 640 libraries for sequencing (NextSeq550, Illumina). As a result, we obtained 522 sequences with very good quality (< 5% of unread [N] nucleotides), which were subsequently submitted to the COVID-19 Data Portal Poland database (Supplementary Table 6). In addition, all sequences were deposited in the European Nucleotide Archive (ENA).

### Analysis of SARS-CoV-2 genetic variability in Poland

**Figure 5:**
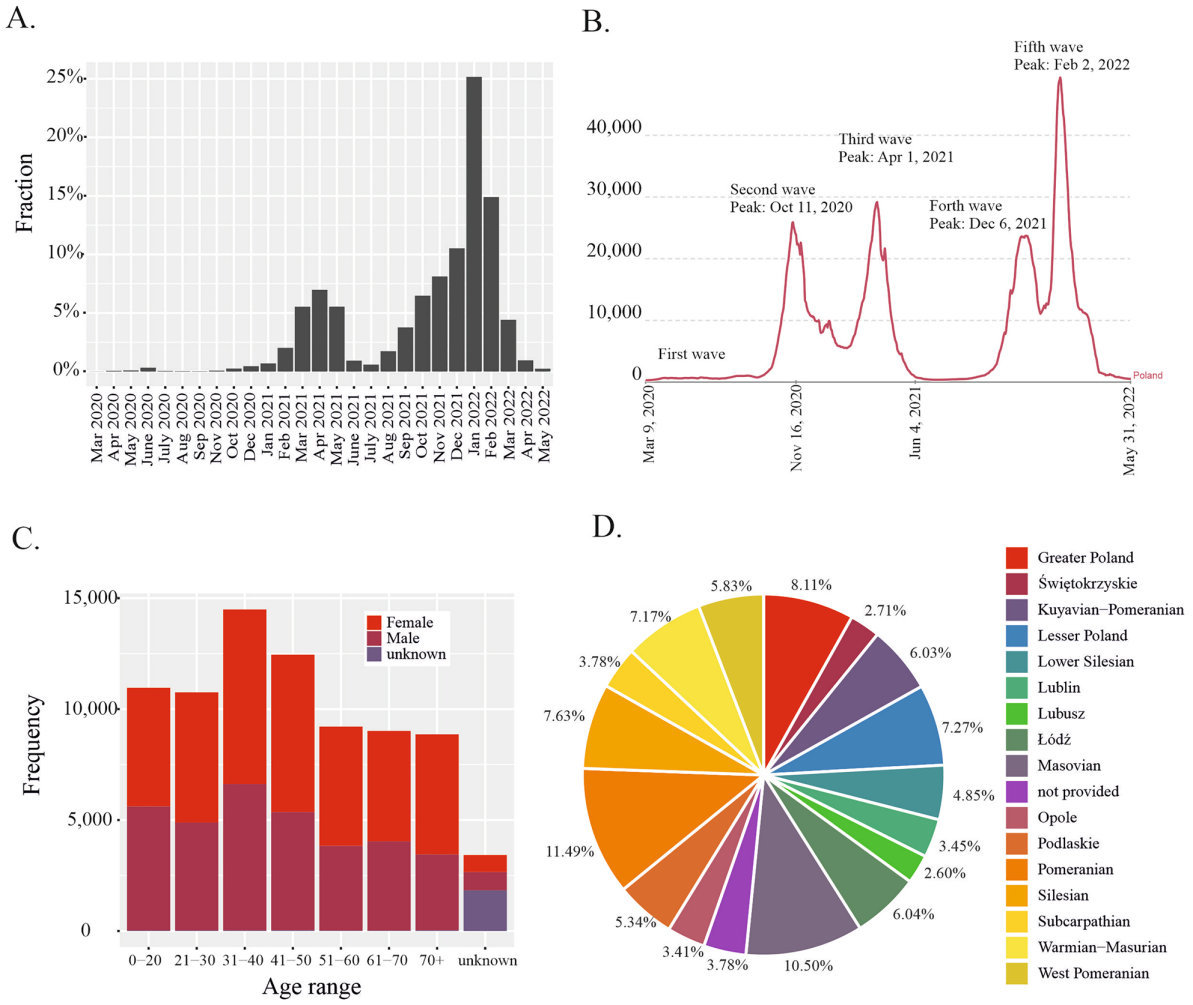
Statistics of the viral genome sequencing data from Poland analyzed in our study. (**A**) Distribution of the sequencing data by sampling time. (**B**) Distribution of the number of daily reported SARS-CoV-2 infections in Poland (7-day rolling average, adapted from Our World In Data under the CC-BY-4.0 license, May 31, 2022). (**C**) Distribution of the sample donors by age and sex. (**D**) Percentage distribution of the analyzed samples per voivodeship.

To analyze the genetic variability of the virus, we used the data generated in our laboratory, as well as 78,657 good-quality SARS-CoV-2 genome sequences from Poland downloaded from the GISAID database (as of May 30, 2022). Most sequences were submitted during the 3rd, 4th, and 5th waves of the COVID-19 pandemic (Fig. 5A, B) and were isolated from middle-aged patients (Fig. 5C). The largest collection of sequencing data was from the Pomeranian (11.49%), Masovian (10.5%), Greater Poland (8.11%), and Silesian (7.63%) voivodeships (Fig. 5D). Generally, we found a strong correlation between the number of submitted sequences and the number of SARS-CoV-2 infections detected in each region (Spearman’s r = 0.76, *p* < 0.05).

**Figure 6:**
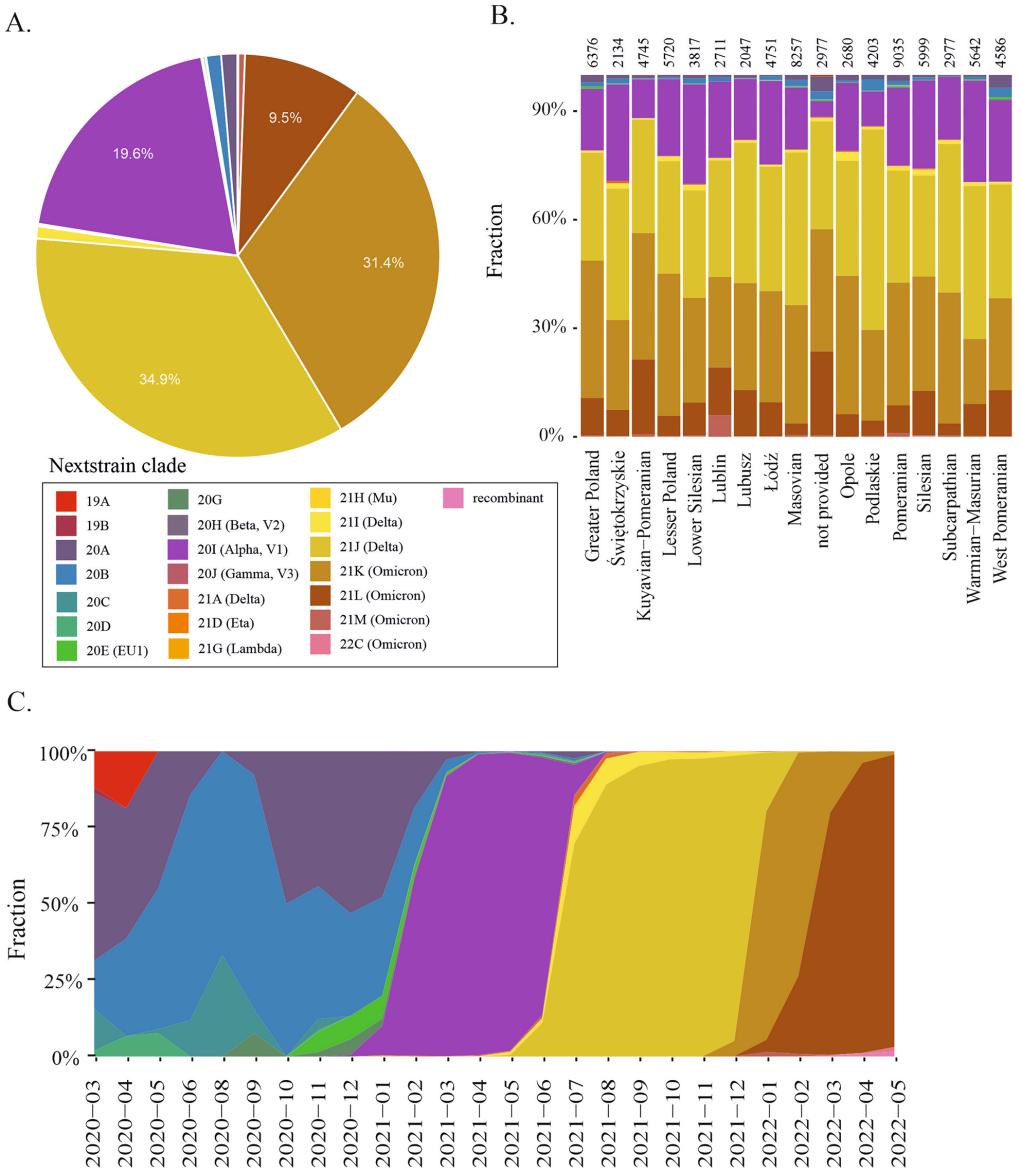
Contribution of individual SARS-CoV-2 clades in the course of COVID-19 in Poland. Nextstrain clade classification was applied. (**A**) Variant distribution in a whole pool of analyzed samples. (**B**) Distribution of SARS-CoV-2 variants by voivodeship. The total number of sequences analyzed is indicated above each bar. (**C**) Distribution of SARS-CoV-2 variants in Poland over time.

The Delta 21J, Omicron 21K, Alpha 20I, and Omicron 21L variants were the most frequent in Poland (Fig. 6A). In each voivodeship, those 4 variants accounted for more than 90% of cases, but their proportions differed (Fig. 6B). The Alpha 20I variant, which circulated between December 2020 and June 2021, was observed in greater proportions in the Świętokrzyskie, Lower Silesian, Warmian-Masurian, and Silesian voivodeships. Nearly 55% of all sequences from the Podlaskie voivodeship were derived from the Delta 21J variant. The Omicron 21K variant accounted for more than 30% of the sequences from Lesser Poland, Opole, Greater Poland, and Subcarpathian. Approximately 20% of sequences from the Kuyavian-Pomeranian voivodeship were identified as Omicron 21L. Both Omicron variants, 21L and 21K, accounted for almost 55% of all variants identified in this region. Interestingly, Lublin was the only voivodeship where a noticeable proportion of the Omicron 21M variant was found.

By analyzing the variant distribution in Poland over time (Fig. 6C), we observed a founding 19A variant in less than 20% of the samples only at the beginning of the pandemic (March–May 2020). The samples sequenced until the end of 2020 mainly contained the 20A and 20B variants and a small proportion of the 20C, 20D, 20E, and 20G variants. Between January and June 2021, almost all the sequences were determined to be the Alpha 20I variant, which was displaced mostly by Delta 21J and small proportions of Delta 21I and Delta 21A. Delta variants circulated slightly longer than did Alpha variants from May 2021 until the end of 2021. Then, the viral population was dominated by Omicron variants. Interestingly, almost 31% of the sequences analyzed in this study were the Omicron 20K variant, which circulated between December 2021 and March 2022. From the end of January 2022, it was slowly displaced by Omicron 21L and disappeared by the end of 2022. Generally, the variant distribution over time in Poland reflected that in Europe (Supplementary Fig. 3B).

### Haplotype network and superspreaders

To identify the virus transmission routes and potential superspreaders, we analyzed haplotype networks built based on the sequence similarity of SARS-CoV-2 variants circulating in Poland during two years of the COVID-19 pandemic. The term 'superspreaders’ usually refers to individuals who become a source of numerous infections and less often to settings, e.g., places and events responsible for extremely effective pathogen dissemination^25^. Due to the high number of sequences available, we generated separate haplotype networks for each pandemic wave (Supplementary Figs. 4–6). Moreover, for waves 3–5, subsampling was performed to reduce the number of sequences (Supplementary Table 7). Otherwise, the networks would have been too dense and impossible to interpret. The subsampling criteria are described in the Methods section.

**Figure 7:**
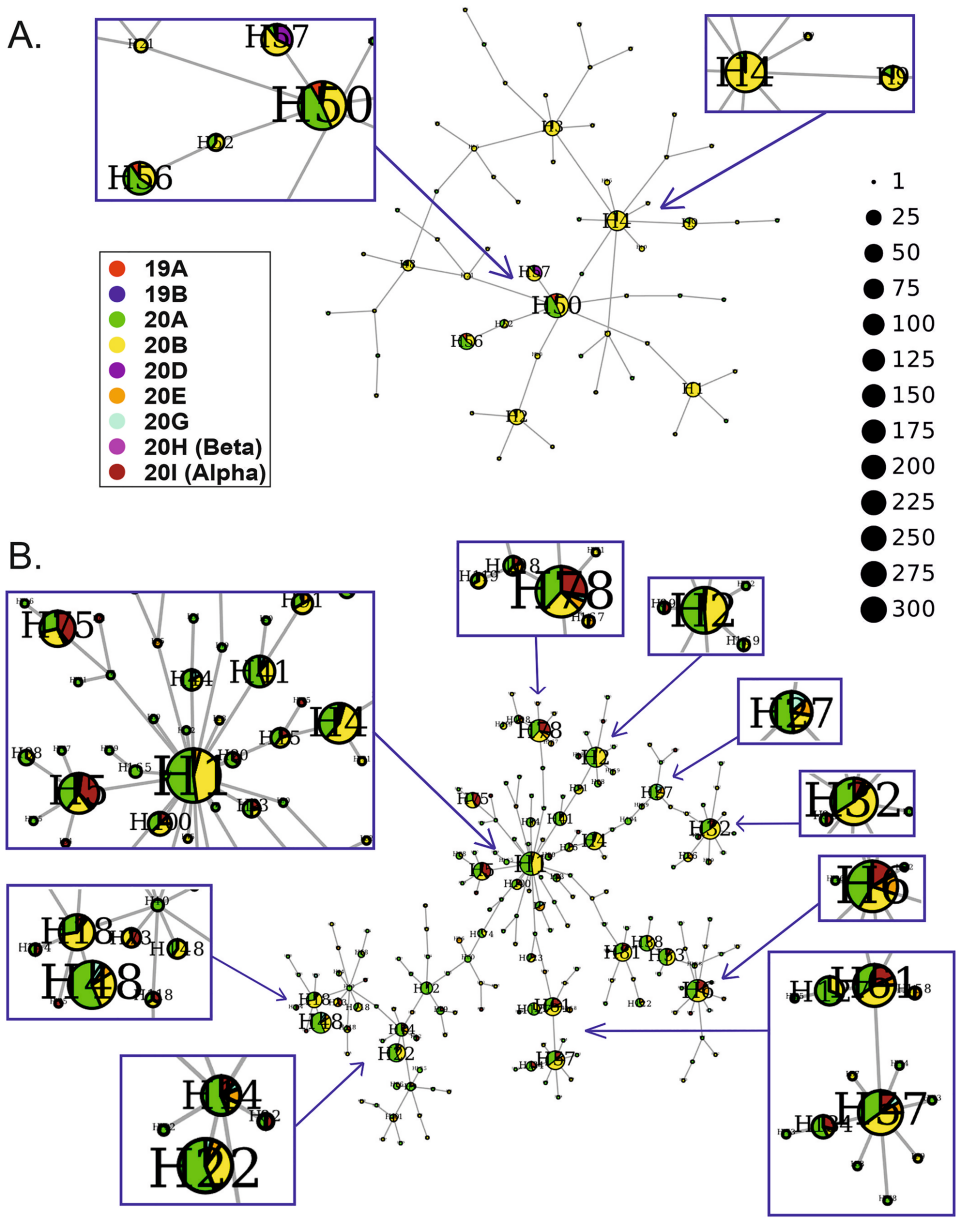
Haplotype networks of SARS-CoV-2 variants identified in the Polish population during the first (**A**) and second (**B**) waves of the COVID-19 pandemic. The superspreaders are enlarged. The sizes of the circles correspond to the numbers of viral sequences included in the nodes.

The 1st wave network was simple and consisted of 58 haplotypes, but only eight nodes were represented by more than 10 cases (Fig. 7A, Supplementary Table 8). Of these, the H56 node contained the earliest SARS-CoV-2 cases (March–April 2020) and can therefore be treated as a symbolic starting point of the pandemic in Poland (Supplementary Fig. 5A). A total of 56% of H56 haplotypes were classified as 20A, 34% as 20B and 9% as 19A variants. Three voivodeships provided the greatest contribution to this node: Masovian, Lesser Poland and Pomeranian (Supplementary Fig. 4A). The largest node, H50, linked closely to H56 (through a smaller H52 node), consisted of 191 haplotypes, most of which belonged to 20A (48%), 20B (41%), and 19A (8%), which was the first SARS-CoV-2 clade (19A). These haplotypes were detected in the Łódź, Pomeranian, Masovian and Lesser Poland voivodeships, but unfortunately, for more than half of the H50 haplotypes, information about the location was not provided. All other nodes, except H57, were composed of 20B variants localized mainly in Greater Poland. This result is likely because IBCH, which was involved in COVID-19 diagnostics in Greater Poland, supplied almost half of the data for the 1st wave in Poland (Supplementary Table 7). Interestingly, in the H57 node, which was assigned mainly to the Pomeranian and Silesian voivodeships, one-third of the variants belonged to the 20D clade.

In the 2nd wave network, built from 1,460 sequences, 185 haplotypes were identified, including 21 represented by more than 10 cases (Fig. 7B, Supplementary Table 9). The largest node, H1, localized in the central part of the plot, reflects the beginning of this wave and corresponds mainly to the Greater Poland voivodeship (Supplementary Figs. 4B and 5B). The H1 node consists of 20A and 20B variants in comparable proportions, similar to its derived nodes H2, H4 and H41. A significant contribution of the 20I (Alpha) variant was observed in nodes H5, H75, H78, H6 and H61. Among the studied locations, potential superspreaders may be found in Greater Poland (H1, H6, H14, H12), Masovian (H53, H57, H61, H4, H75), West Pomeranian (H48, H41, H38), and Świętorzyskie (H81, H2, H4). The highest proportion of the 20I (Alpha) variant (> 25%) was observed in H5 (corresponding mainly to Warmian-Masurian) and H75 (shared by Masovian and Podlaskie).

**Figure 8:**
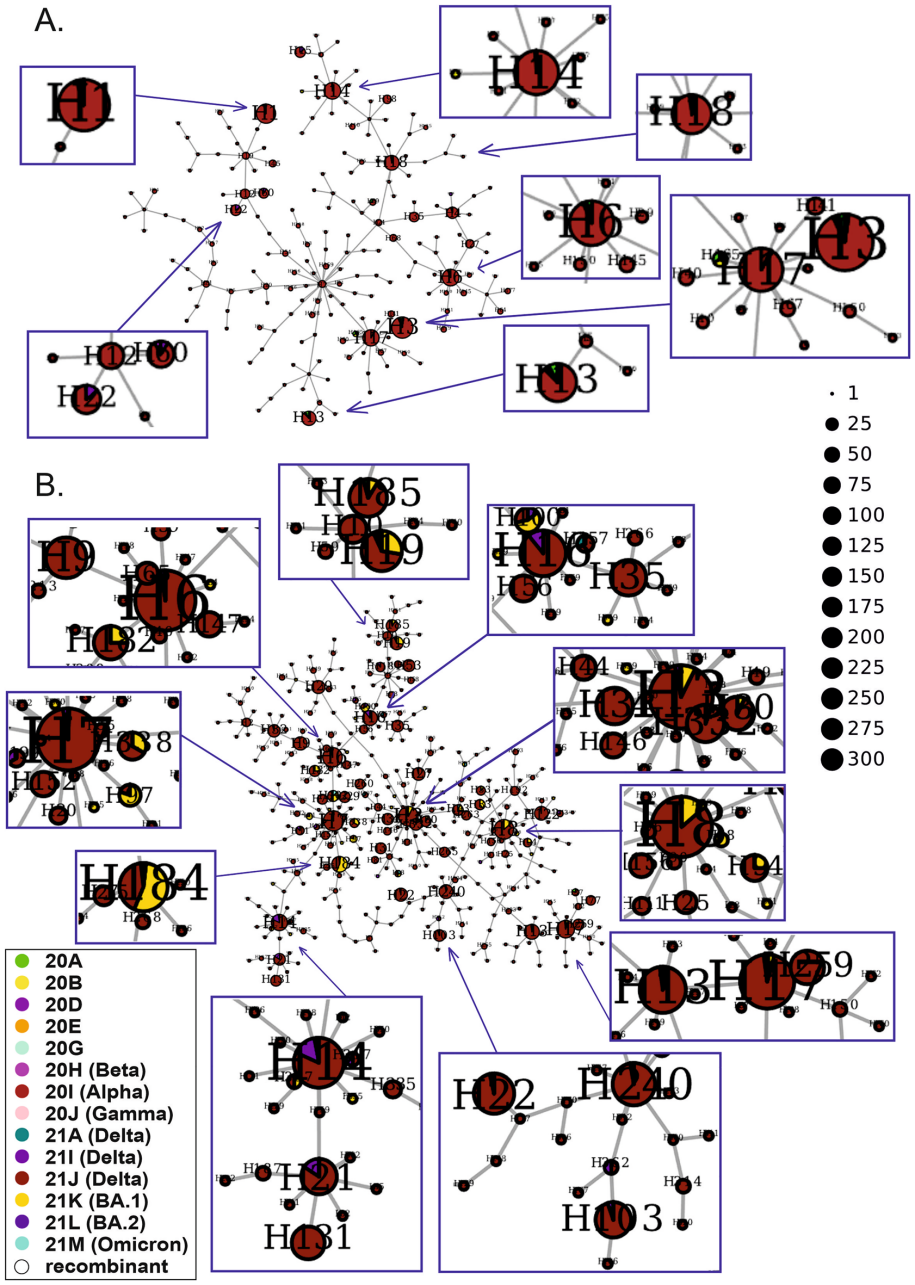
Haplotype networks of SARS-CoV-2 variants identified in the Polish population during the third (**A**) and fourth (**B**) waves of the COVID-19 pandemic. The superspreaders are enlarged. The sizes of the circles correspond to the numbers of viral sequences included in the nodes.

The haplotype network generated for wave 3 (first half of 2021) consisted of 187 nodes, but only 13 contained more than 10 cases (Fig. 8A, Supplementary Table 10). The whole network is entirely dominated by the 20I (Alpha) variant (989 cases out of 1050 subsampled), which is consistent with the plot in Fig. 6C. The earliest cases were part of node H6, assigned to the Lower Silesian, Łódź, Świętokrzyskie, Greater Poland, Silesian and Warmian-Masurian voivodeships (Supplementary Figs. 4C and 5C). Similarly, two larger nodes (H3 and H1) were balanced mixtures of different voivodeships, demonstrating that the pandemic spread equally throughout the entire country. However, in some hubs, particular voivodeships predominated, which indicates the presence of potential superspreaders: Pomeranian (H13, H22, H60, H14), Lesser Poland (H18, H68), Lower Silesian (H4, H6, H27), Masovian (H17, H35, H15), Świętkorzyskie (H12) and Łódź (H2).

The wave 4 network was quite dense (362 nodes, including 39 with at least 10 cases) and dominated by the 21J (Delta) variant (1903 out of 2107, Fig. 8B, Supplementary Table 11). Another variant that contributed to this wave, although to a much lesser extent, was 21K (BA.1). This variant constituted more than half of the haplotypes in the H184 node, assigned mainly to Masovian and Lesser Poland (Supplementary Fig. 4D). A small number of cases of 21I (Delta) was also detected, particularly at node H14 (Silesian, Lesser Poland, Opole). Large nodes with significant contributions from particular voivodeships may be considered potential superspreaders, e.g., H16 (100% Silesian), H122 (100% Podlaskie), H7 (shared equally by Łódź and Greater Poland), H3 and H13 (Warmian-Masurian, 52% and 44%, respectively).

**Figure 9:**
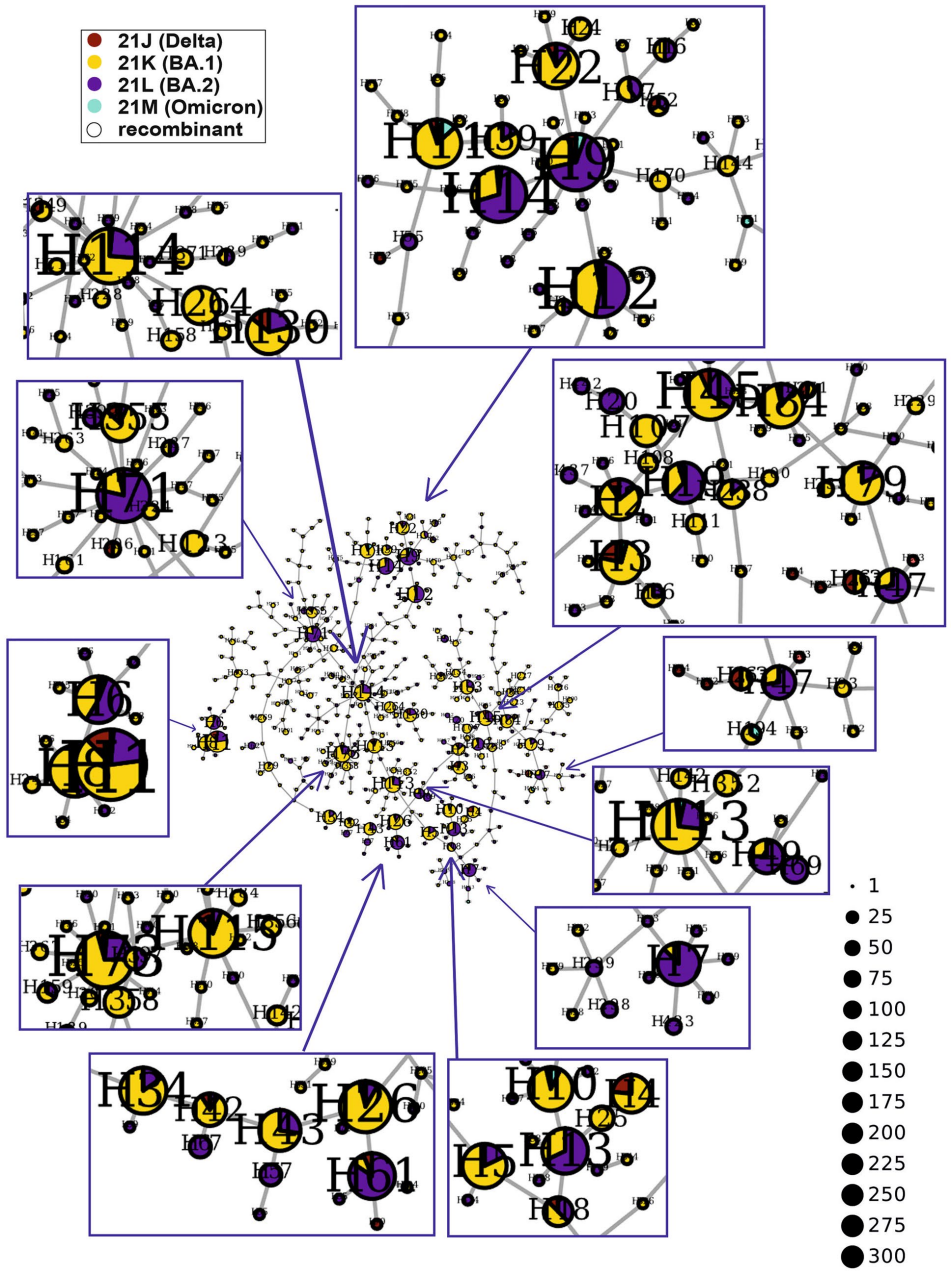
Haplotype network of SARS-CoV-2 variants identified in the Polish population during the fifth wave of the COVID-19 pandemic. The superspreaders are enlarged. The sizes of the circles correspond to the numbers of viral sequences included in the nodes.

During wave 5, two variants, previously called Omicron, i.e., 21K (BA.1, 63%) and 21L (BA.2, 31%), predominated, with small contributions of 21J (Delta, < 5%) and 21M (Omicron, 1%). The whole network consisted of 445 nodes, 36 of which were represented by more than 10 cases (Fig. 9, Supplementary Table 12). Among them, H7, H61, H9, H14, H71 and H13, dated to March–April 2022 (Supplementary Fig. 5E), were dominated by the 21L (BA.2) clade. Most nodes were divided between different voivodeships (Supplementary Fig. 4E). However, in some nodes, specific voivodeships predominated, e.g., Silesian (100% of H39, 76% of H26, 70% of H71, 64% of H45), Greater Poland (68% of H5, 63% of H7, 61% of H3, 50% of H6, 48% of H2), Kuyavian-Pomeranian (65% of H13, 64% of H130, 51% of H83, 49% of H12, 19% of H1), Lublin (64% of H9, 58% of H11, 48% of H8), Warmian-Masurian (56% of the largest node, H1, and 26% of H12), Subcarpathian (74% of H10, 42% of H11) and Opole (61% of H34).

## Discussion

The outbreak of the COVID-19 pandemic has changed the world irreversibly because it occurred in the era of the internet and global information exchange. On the one hand, high population density and commonly available long-distance travel enabled the spread of the virus. On the other hand, the development of science and technology allowed for an immediate response through massive testing and vaccinations. After the pandemic passed, it was determined that many factors influenced the spread of the virus and the course of the pandemic, including local restriction policies, testing and vaccination strategies, demographic features, sociobehavioral aspects, and the level of health care in each country^26^.

The response to the COVID-19 pandemic varied across countries. For example, in the beginning of the pandemic, the governments of the UK, the US, and Sweden decided to pursue the herd natural immunity strategy, which was criticized worldwide^27^. A high number of reinfections revealed that this strategy was not optimal in the case of SARS-CoV-2^28,29^. Analysis of the first year of the pandemic in Nordic countries (Denmark, Norway, Iceland, Sweden, and Finland) showed that the recommendation-based model in Sweden rather than legislation-based restriction models led to increased SARS-CoV-2 infections^30^. Long-term observation in the UK also confirmed that the most efficient strategy was based on restrictions^31^. Travel restrictions and border migration control in the EU prevented the virus from spreading^32^. In Poland, soon after the first SARS-CoV-2 infection was reported, the government started to impose restrictions, including social distancing, travel limitations, and massive testing^33^. The restrictions in Poland were relaxed for the first time in June-October 2020. At the same time, restrictions were relaxed in Czechia, Lithuania, and Slovakia. When the second coronavirus wave hit approximately November 2020, a drastic increase in the number of excess deaths occurred in those four countries (Fig. 1E). Therefore, it can be assumed that restriction relaxation during the summer holidays contributed to the sudden increase in fatal infections in Poland and neighboring countries.

Despite relatively low numbers of infections per million people, Poland had a high death rate, comparable to that of the US, UK, and Italy, countries that reported more COVID-19 cases. The simplest explanation is that the actual number of cases in Poland was underestimated due to an insufficient number of tests performed. However, a low ratio of the number of tests to the number of detected cases was also observed in Germany, Sweden, and Lithuania. These countries had much lower CFRs than Poland. The observed contrast might result from differences in health care efficiency in particular countries. A report published in 2021^34^ (based on Organization for Economic Cooperation and Development (OECD) data (stats.oecd.org)) showed that Poland ranked third from the bottom in terms of spending on health care and expenditures per capita. In 2019, expenditures on health care in Poland amounted to 4.3% of gross domestic product (GDP), which was much lower than the European average (6.53%). The highest ranking country was Germany (9.9% GDP), followed by France (9.4% GDP) and Sweden (9.3% GDP). It is worrying that health—related expenditures in Poland as a portion of GDP, after an increase in 2017, have been decreasing since 2018. The consequences might be severe: limited access to medical care, delayed medical intervention, and untreated chronic diseases. In our opinion, comorbidities could have contributed to the higher COVID-19-related mortality in Poland.

Mass vaccinations of the population helped to address the pandemic. Multiple studies have shown that even if vaccination does not prevent the possibility of viral infection, it affected the course of COVID-19 and contributed to decreased mortality^35–39^. Among the European countries, Poland, Hungary, Romania, and Bulgaria made no progress in reducing the number of COVID-19-related deaths^40^. The study showed a strong reverse correlation between mortality and the primary vaccination rate. Consistently, our data revealed a strong negative correlation between the CFR and the percentage of the vaccinated population in Poland. Notably, Bulgaria, Romania, and Slovakia had lower vaccination rates and more excess deaths per 100,000 people than Poland^3^. Israel, the first country worldwide to launch a massive vaccination strategy, exhibited the opposite results. In March 2021, 50% of its population was vaccinated (Fig. 1G)^41^. Despite the relatively high number of cases, a low number of COVID-19-related deaths and excess deaths were noted in Israel (Fig. 1A–E). Nevertheless, Israel had one of the lowest CFRs worldwide^3^. This finding indicates that the faster a vaccination is implemented, the better the prevention.

Interestingly, Kim et al. analyzed the incidence and mortality of COVID-19 in 107 countries worldwide^42^. They considered many factors, including socioeconomic and demographic features, geography, health care capacity, and health behaviors. They found that COVID-19 incidence and the CFR were influenced mainly by ethnicity and that East Asia was the least influenced by the pandemic. The authors concluded that Asian populations might be more resistant to SARS-CoV-2, as they have likely encountered similar pathogens in the past.

Regarding regional differences in Poland, the greatest total number of cases and deaths were observed in the Masovian and Silesian voivodeships. Warsaw, the capital of Poland and the largest and most populated city in the country, is located in the Masovian voivodeship. Warsaw is also a large labor market and international transfer hub and a potential source of new infections. Silesian is one of the smallest voivodeships in Poland but is the most urbanized region in the country, with the highest population density^43^ and severe air pollution. Some research has noted the influence of air pollution on susceptibility to viral infections, including SARS-CoV-2^44–46^. An association between air pollution and COVID-19 mortality and morbidity was also observed in Poland^47^. Our data (Supplementary Table 5) showed that the highest CFR was found in Subcarpathian, Świętokrzyskie, and Lublin, regions that are less polluted and sparsely populated. In this case, insufficient vaccination seems to be the main reason for the high CFR, as Subcarpathian and Lublin are among the three voivodeships with the lowest vaccination rates.

Since the discovery of the first SARS-CoV-2 genome sequence, many subsequent variants with different transmission potentials and virulence levels have been described^48–52^. The worldwide variant evolution over time^24,53^ shows that new SARS-CoV-2 variants occasionally appeared and spread more successfully (Supplementary Fig. 3). For example, in Europe, the Alpha variant circulating between January and July 2021 displaced earlier variants (19A, 20A, 20B, 20E) and was further displaced by Delta (circulating June-December 2021) (Fig. 5B). In turn, the Delta variant was effectively replaced by Omicron 21K and its variants (21L, 21M, 22A, 22B, and 22C). The distribution of variants over time was generally similar in Europe and worldwide (Supplementary Fig. 3C). The differences occurred in autumn and winter 2020, when the 20E (EU1) variant was spreading mainly in Europe, and in spring and summer 2021, when a greater proportion of the Gamma variant (20J) (Supplementary Fig. 3C) was observed in the world than in Europe. At the same time, a lower percentage of the 20I Alpha VI variant and a slightly greater percentage of beta (20H) and lambda (21G) variants were observed worldwide than in Europe. Approximately November 2021, the Omicron variant (21K) efficiently displaced other variants, and since then, Omicron subvariants (21L, 22A, 22B and 22C) have circulated throughout the world (Supplementary Fig. 3C).

Our analysis of the viral genome sequencing data showed that the overall variant distribution in Poland over time did not differ from that in the rest of Europe. However, we found some regional differences in Poland. For example, the Delta variant 21J was responsible for ~ 55% of infections in Podlaskie and was also very frequent in the Subcarpathian, Świętokrzyskie, Lubusz, Masovian, and Warmian-Masurian voivodeships. All of them except Masovian had very high CFRs and low vaccination rates. Therefore, it cannot be excluded that the molecular features of Delta 21J, together with the low vaccination rate, contributed to higher mortality in these regions. Indeed, the Delta variant possesses multiple mutations in the Spike gene, making the virus 60% more transmissible than the Alpha variant and causing a more severe course of COVID-19^54,55^.

The haplotype network analysis of sequences obtained at the beginning of the pandemic (waves 1 and 2) may be biased by the small number of reported cases and unequal representation of sequenced samples for all regions of Poland. The haplotype networks for waves 4 and 5 are denser and more uniformly dispersed, which showed that in the second half of 2021, the pathogen was gradually, widely and evenly transmitted throughout the country. Nevertheless, some regional and temporal trends indicated the presence of potential superspreaders, particularly in Greater Poland (waves 1, 2, 4 and 5), Silesian and Warmian-Masurian (waves 4 and 5), Pomeranian and Lesser Poland (wave 3), Masovian and West Pomeranian (wave 2), and Kuyavian-Pomeranian and Lublin (wave 5) voivodeships. These results reflect the dynamics of the COVID-19 pandemic.

In conclusion, our research enabled us to demonstrate the relationships between various factors that could have been responsible for the course of the COVID-19 pandemic in Poland and other countries. Overall, the COVID-19 experience has become an invaluable resource for understanding infectious diseases and viral evolution. Learning from multiple studies on COVID-19 should allow us to better prepare for future pandemics.

### Supplementary Information

Supplementary Information 1.

Supplementary Tables.

Supplementary Figures.

## Data Availability

The data sequenced in our laboratory are available here: https://www.ebi.ac.uk/ena/browser/view/PRJEB39761 (sample IDs used in the analysis are listed in the first column of Supplementary Table 6). The data downloaded from GISAID, with the identifier EPI_SET_231114ua, are available at 10.55876/gis8.231114ua. All genome sequences and associated metadata in this dataset are published in GISAID’s EpiCoV database (https://www.epicov.org/epi3/frontend#256835). EPI_SET_231114ua is composed of 78,674 individual genome sequences. The collection dates ranged from 2020-03-03 to 2022-05-23. The data were collected in Poland. All sequences in this dataset were compared to those of hCoV-19/Wuhan/WIV04/2019 (WIV04), the official reference sequence employed by GISAID (EPI_ISL_402124, https://gisaid.org/WIV04).
