# Supplementary material for: The landscape of the COVID-19 pandemic in Poland emerging from epidemiological and genomic data
Source: Sci Rep. 2024 Jun 22;14:14416. doi: 10.1038/s41598-024-65468-5 (PMC11193717; doi:10.1038/s41598-024-65468-5)
Supplement: Supplementary file 1 — Supplementary Information 1. [file 41598_2024_65468_MOESM1_ESM.pdf]

SUPPLEMENTAL TABLE

Data Availability

GISAID Identifier:	EPI_SET_231114ua
doi:	10.55876/gis8.231114ua

All genome sequences and associated metadata in this dataset are published in GISAID's EpiCoV database. To view the contributors of each individual sequence with details such as accession number, Virus name, Collection date, Originating Lab and Submitting Lab and the list of Authors, visit 10.55876/gis8.231114ua

Data Snapshot

- EPI_SET_231114ua is composed of 78,674 individual genome sequences.
- The collection dates range from 2020-03-03 to 2022-05-23;
- Data were collected in 1 countries and territories;
- All sequences in this dataset are compared relative to hCoV-19/Wuhan/WIV04/2019 (WIV04), the official reference sequence employed by GISAID (EPI_ISL_402124). Learn more at https://gisaid.org/WIV04.
