# Supplementary material for: The landscape of the COVID-19 pandemic in Poland emerging from epidemiological and genomic data
Source: Sci Rep. 2024 Jun 22;14:14416. doi: 10.1038/s41598-024-65468-5 (PMC11193717; doi:10.1038/s41598-024-65468-5)
Supplement: Supplementary file 3 — Supplementary Figures. [file 41598_2024_65468_MOESM3_ESM.pdf]

Supplementary Figures

The landscape of the COVID-19 pandemic in Poland emerging from epidemiological and genomic data

Barbara Mirska[1], Michal Zenczak[1], Katarzyna Nowis[1], Ireneusz Stolarek[1], Jan Podkowiński[1], Magdalena Rakoczy[1], Małgorzata Marcinkowska-Swojak[1], Natalia Koralewska[1], Paweł Zmora[1], Elżbieta Lenartowicz Onyekaa[1], Marcin Osuch[1], Katarzyna Łasińska[2], Jadwiga Kuczma-Napierała[2], Marcelina Jaworska[3], Łukasz Madej[4], Marzena Ciechomska[5], Aleksander Jamsheer[6,7], Krzysztof Kurowski,[1] Marek Figlerowicz[1], Luiza Handschuh[1*]

1 Institute of Bioorganic Chemistry Polish Academy of Sciences, Poznan, Poland
2 Provincial Sanitary and Epidemiology Station in Poznan, Poland
3 Baptism of Poland Memorial Hospital, Gniezno, Poland
4 Regional Science and Technology Center, Podzamcze, Poland
5 National Institute of Geriatrics, Rheumatology and Rehabilitation, Warsaw, Poland
6 Poznan University of Medical Sciences, Department of Medical Genetics, Poznan, Poland
7 Centers for Medical Genetics GENESIS, Poznan, Poland
* corresponding author: luizahan@ibch.poznan.pl

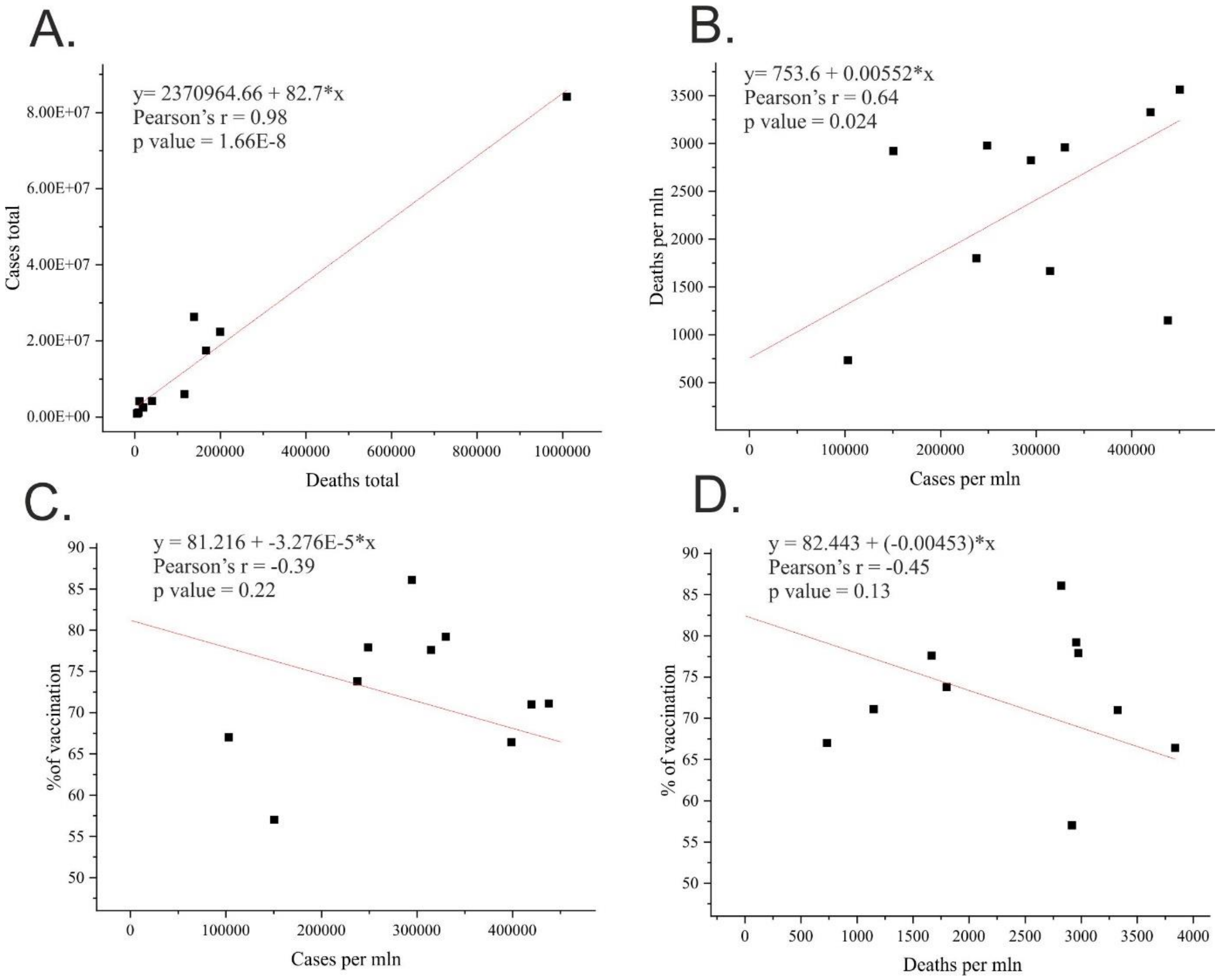

Supplementary Figure 1. Linear regression plots with fitted regression lines, presenting correlations between the following variables in the selected countries (listed in Supplementary Table 1): **A.** Total number of deaths due to COVID-19 and total number of COVID-19 cases. **B.** Total numbers of cases and deaths normalized per 1 million people. **C.** Total number of cases normalized per 1 million people and percentage of vaccinated population. **D**. Total number of deaths normalized per 1 million people and percentage of the population that was vaccinated. Regression equations and Pearson's r coefficients are indicated on each plot.

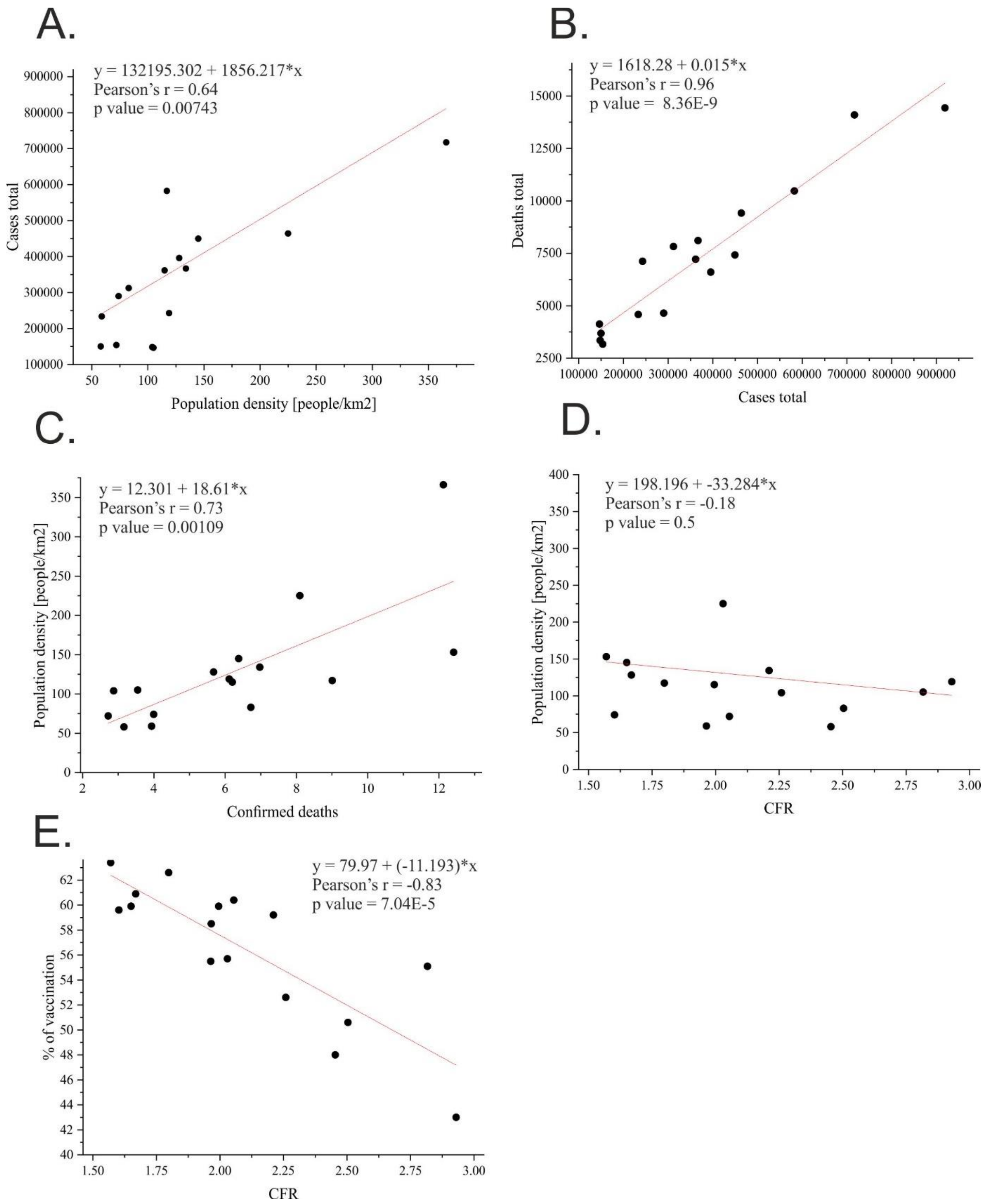

Supplementary Figure 2. Linear regression plots with fitted regression lines, presenting correlations between the following variables in the voivodeships of Poland (listed in Supplementary Table 3): **A.** Population density [people/km2] and total number of COVID-19 cases. **B.** Total number of COVID-19 cases and total number of deaths due to COVID-19. **C.** Number of confirmed deaths (presented as a percentage of total number of deaths in the country) and population density [people/km2]. **D**. CFR (Case Fatality Rate) and population density [people/km^2]. **E**. CFR and percentage of the population that was vaccinated. Regression equations and Pearson's r coefficients are indicated on each plot.

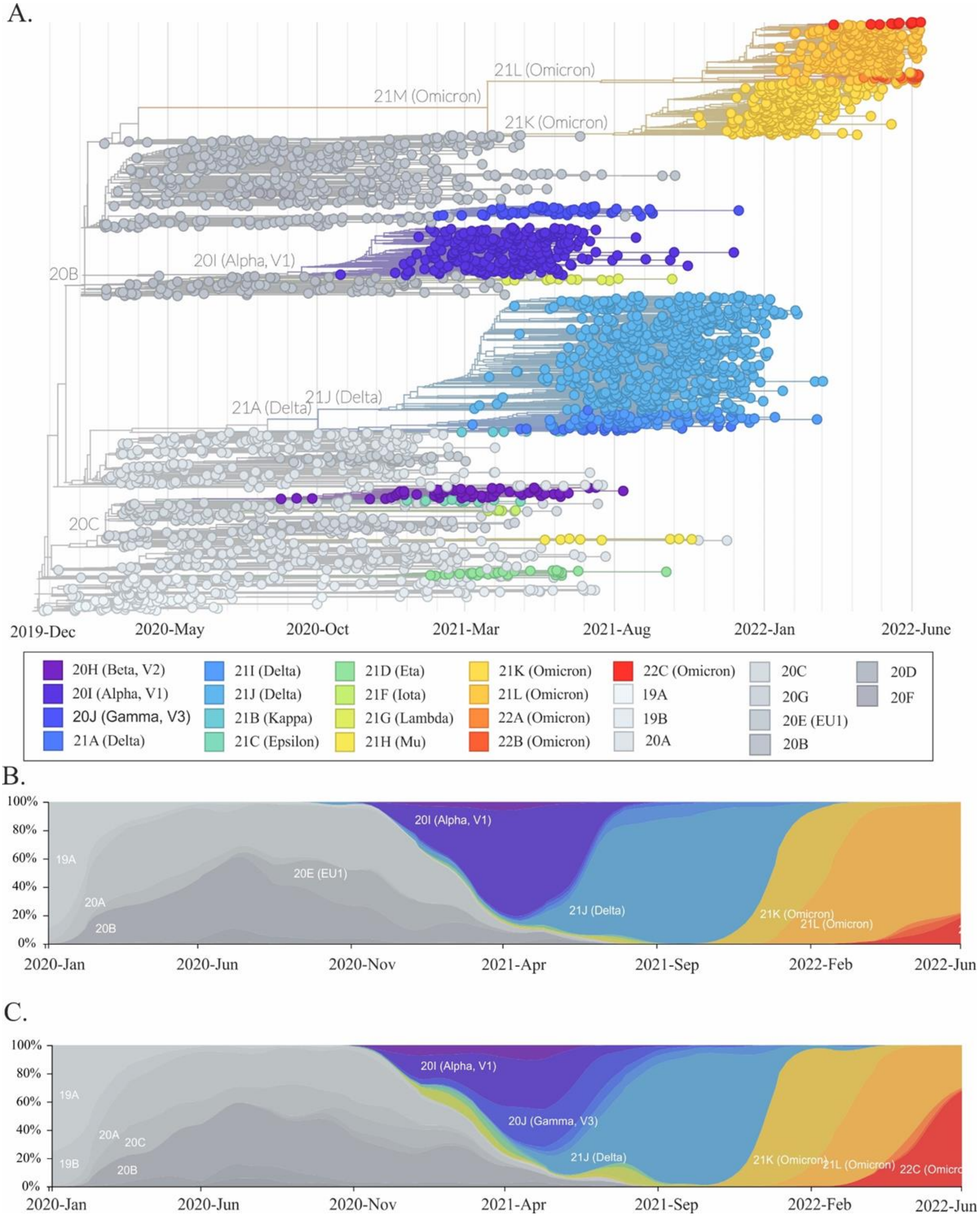

Supplementary Figure 3. SARS-CoV-2 evolution during COVID-19 pandemic – global scale. A. Phylogenetic tree of the SARS-CoV-2 virus with particular clades highlighted. The phylogeny was calculated based on 3081 genomes sampled between December 2019 and the end of May 2022 worldwide. **B.** Frequencies of SARS-CoV-2 variants circulating over time in Europe. **C.** Frequencies of SARS-CoV-2 variant circulating over time worldwide. All figures were downloaded from https://gisaid.org/phylodynamics/global/nextstrain/) and used under a CC-BY-4.0 license.

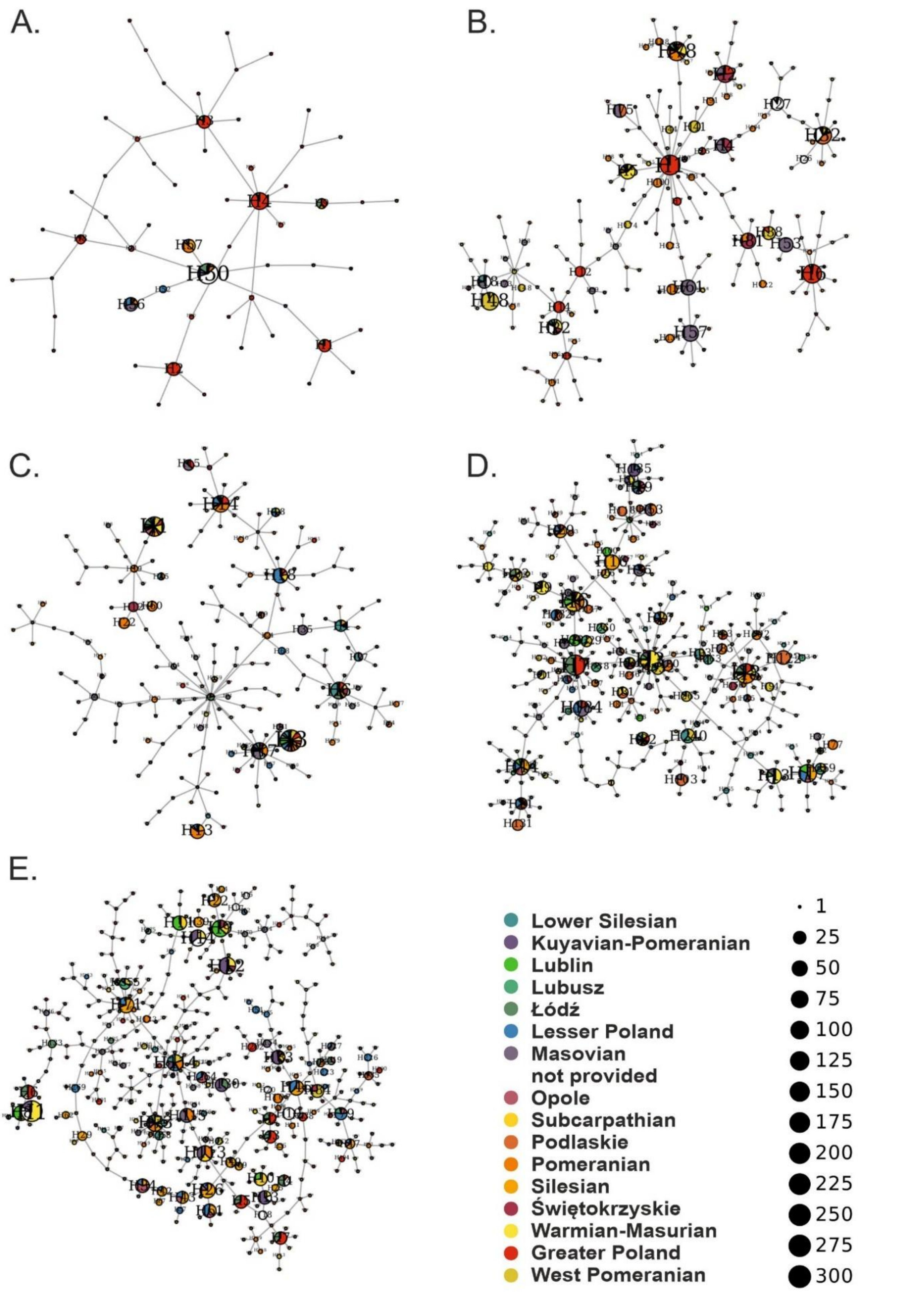

Supplementary Figure 4. Haplotype networks of SARS-CoV-2 variants identified in Poland population during 2 years of COVID-19 pandemic. Voivodeships where haplotypes were detected are colored. The sizes of the circles correspond to the numbers of viral sequences included in the nodes. **A.** Wave 1. **B.** Wave 2. **C.** Wave 3. **D.** Wave 4. **E.** Wave 5.

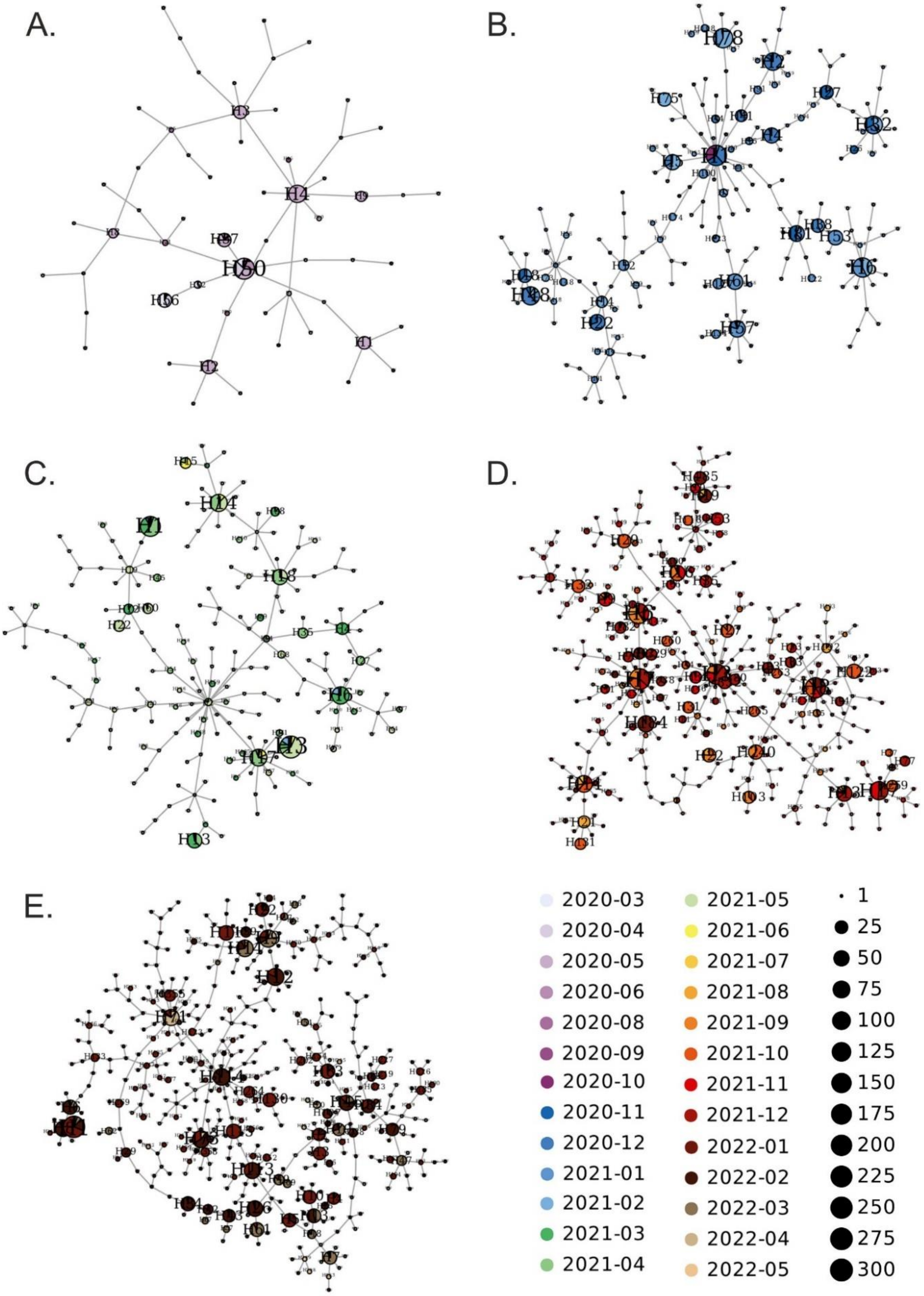

Supplementary Figure 5. Haplotype networks of SARS-CoV-2 variants identified in Poland population during 2 years of COVID-19 pandemic. The months in which haplotypes were detected are colored. The sizes of the circles correspond to the numbers of viral sequences included in the nodes. **A.** Wave 1. **B.** Wave 2. **C.** Wave 3. **D.** Wave 4. **E.** Wave 5.

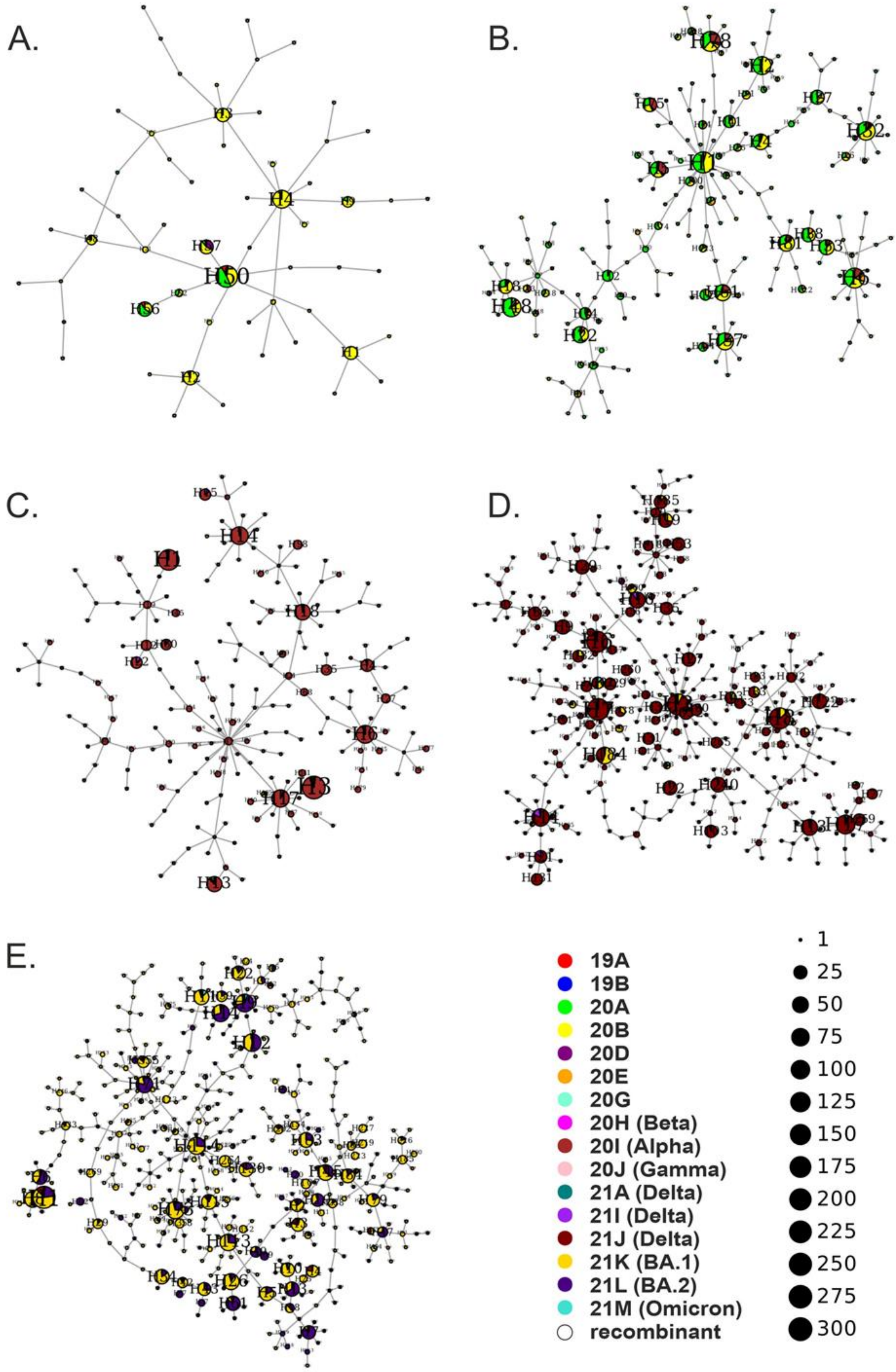

Supplementary Figure 6. Haplotype networks of SARS-CoV-2 variants identified in Polish population during 2 years of COVID-19 pandemic. Detected variants are colored. The sizes of the circles correspond to the numbers of viral sequences included in the nodes. **A.** Wave 1. **B.** Wave 2. **C.** Wave 3. **D.** Wave 4. **E.** Wave 5.
